# Access to Innovative Hematological Treatments in Morocco: Delays, Barriers, and a Public Health Concern

**DOI:** 10.7759/cureus.102206

**Published:** 2026-01-24

**Authors:** Monsif Fadi, Fatima Azzahra Lahlou, Nouama Bouanani

**Affiliations:** 1 Oncopathology, Center for Doctoral Studies (CEDoc), Cancer Biology and Environment Laboratory, Faculty of Medicine, Mohammed VI University of Health Sciences (UM6SS), Casablanca, MAR; 2 Biochemistry, Health and Environment Laboratory, Ain Chock Faculty of Sciences, Hassan II University, Casablanca, MAR; 3 Biochemistry, Mohammed VI National Laboratory, Mohammed VI University of Health Sciences (UM6SS), Casablanca, MAR; 4 Hematology, Faculty of Medicine, Mohammed VI University of Health Sciences (UM6SS), Casablanca, MAR

**Keywords:** access to care, chronic lymphocitic leukemia, morocco, public health policy, targeted therapies

## Abstract

Therapeutic advances in hematology, particularly the introduction of targeted agents, have substantially improved outcomes of patients with chronic lymphocytic leukemia (CLL). However, access to these innovative treatments remains uneven across regions, especially in middle-income countries. In Morocco, limited availability of Bruton tyrosine kinase and BCL-2 inhibitors, delays in regulatory approval, and financial and reimbursement barriers continue to shape real-world treatment strategies. As a result, many patients still receive conventional chemotherapy-based regimens despite international guideline recommendations favoring chemotherapy-free approaches. This editorial highlights the current gaps between evidence-based standards and clinical practice in Morocco, reviews available epidemiological data, and discusses the regulatory and structural obstacles affecting timely access to novel therapies. Addressing these disparities is essential to improve equity of care and ensure that therapeutic progress translates into meaningful clinical benefit for all patients with CLL.

## Editorial

Therapeutic advances in hematology, particularly the development of targeted agents and pathway-specific inhibitors, have significantly improved outcomes in many hematologic malignancies. In chronic lymphocytic leukemia (CLL), Bruton tyrosine kinase (BTK) inhibitors and BCL-2 inhibitors have transformed disease management, offering durable disease control with better tolerability compared with conventional chemoimmunotherapy. Despite these advances, access to innovative hematological treatments remains limited in many middle-income countries, including Morocco, resulting in disparities between evidence-based recommendations and real-world practice [1,2].

Epidemiological data on CLL in Morocco remain scarce due to the absence of a national population-based registry for hematological malignancies. Available evidence is mainly derived from regional registries and hospital-based studies. A multicenter retrospective study from Eastern Morocco reported that CLL represented approximately 5.8% of all hematological malignancies, with a male predominance and a median age at diagnosis of 67 years, consistent with global epidemiological patterns [3]. The Casablanca Population-Based Cancer Registry indicated that hematological malignancies had an overall incidence of 8.4 cases per 100,000 inhabitants between 2004 and 2007, although CLL-specific incidence was not separately reported [4]. Together, these findings highlight that CLL constitutes a tangible clinical burden in Morocco and underline the need for comprehensive national epidemiological surveillance.

Despite the widespread adoption of chemotherapy-free regimens in international guidelines, real-world treatment patterns in Morocco remain constrained by limited access to targeted therapies. In our institution, approximately 70 % of patients with CLL continue to receive conventional chemotherapy or chemoimmunotherapy as first-line therapy, largely due to restricted availability of BTK and BCL-2 inhibitors, delays in reimbursement, and financial barriers. These limitations mean that treatment decisions are often influenced more by available resources than by disease biology or patient-specific risk factors, reflecting the persistent gap between international recommendations and local practice.

Regulatory and logistical barriers further exacerbate these challenges. Even when innovative therapies are approved internationally, substantial delays ranging from two to over five years can occur before local authorization and reimbursement. Some therapies remain unavailable years after approval by major regulatory agencies, preventing timely patient access to standard-of-care treatments [5].

Addressing these challenges requires a multifaceted approach. Aligning national regulatory timelines with international approvals, integrating innovative hematological therapies into reimbursement frameworks, and prioritizing value-based decision-making are crucial steps toward equitable care. In CLL, where targeted therapies are now central to standard management, delayed or unequal access risks widening disparities in patient outcomes. Consequently, timely and affordable access to innovative hematological treatments should be considered both a strategic component of national cancer control policies and a measurable indicator of health system performance.

Finally, future studies should aim to generate robust, population-based epidemiological data on CLL in Morocco and assess the impact of limited access to targeted therapies on patient survival and quality of life. Comparative analyses with other middle-income countries could provide insights to inform policy and guide resource allocation, ensuring that therapeutic advances benefit all patients equitably.
